# Quantification of [^18^F]afatinib using PET/CT in NSCLC patients: a feasibility study

**DOI:** 10.1186/s13550-020-00684-4

**Published:** 2020-08-17

**Authors:** E. A. van de Stadt, M. Yaqub, A. A. Lammertsma, A. J. Poot, P. R. Schober, R. C. Schuit, E. F. Smit, I. Bahce, N. H. Hendrikse

**Affiliations:** 1 Department of Pulmonology, Amsterdam UMC location VUmc, Amsterdam, the Netherlands; 2 Cancer Center Amsterdam, Amsterdam UMC, Amsterdam, the Netherlands; 3 Department of Radiology and Nuclear Medicine, Amsterdam UMC location VUmc, Amsterdam, the Netherlands; 4 Department of Anesthesiology, Amsterdam UMC location VUmc, Amsterdam, the Netherlands; 5 Department of Thoracic Oncology, Netherlands Cancer Institute, Amsterdam, the Netherlands; 6 Department of Clinical Pharmacology and Pharmacy, Amsterdam UMC location VUmc, Amsterdam, the Netherlands

**Keywords:** [^18^F] Afatinib, NSCLC, EGFR TKI, PET/CT, Targeted therapy, Lung cancer

## Abstract

**Introduction:**

Only a subgroup of non-small cell lung cancer (NSCLC) patients benefit from treatment using epidermal growth factor receptor (EGFR) tyrosine kinase inhibitors (TKI) such as afatinib. Tumour uptake of [^18^F]afatinib using positron emission tomography (PET) may identify those patients that respond to afatinib therapy. Therefore, the aim of this study was to find the optimal tracer kinetic model for quantification of [^18^F]afatinib uptake in NSCLC tumours.

**Methods:**

[^18^F]Afatinib PET scans were performed in 10 NSCLC patients. The first patient was scanned for the purpose of dosimetry. Subsequent patients underwent a 20-min dynamic [^15^O]H_2_O PET scan (370 MBq) followed by a dynamic [^18^F]afatinib PET scan (342 ± 24 MBq) of 60 or 90 min. Using the Akaike information criterion (AIC), three pharmacokinetic plasma input models were evaluated with both metabolite-corrected sampler-based input and image-derived (IDIF) input functions in combination with discrete blood samples. Correlation analysis of arterial on-line sampling versus IDIF was performed. In addition, perfusion dependency and simplified measures were assessed.

**Results:**

Ten patients were included. The injected activity of [^18^F]afatinib was 341 ± 37 MBq. Fifteen tumours could be identified in the field of view of the scanner. Based on AIC, tumour kinetics were best described using an irreversible two-tissue compartment model and a metabolite-corrected sampler-based input function (Akaike 50%). Correlation of plasma-based input functions with metabolite-corrected IDIF was very strong (*r*^2^ = 0.93). The preferred simplified uptake parameter was the tumour-to-blood ratio over the 60- to 90-min time interval (TBR_60–90_). Tumour uptake of [^18^F]afatinib was independent of perfusion.

**Conclusion:**

The preferred pharmacokinetic model for quantifying [^18^F]afatinib uptake in NSCLC tumours was the 2T3K_vb model. TBR_60–90_ showed excellent correlation with this model and is the best candidate simplified method.

**Trial registration:**

https://eudract.ema.europa.eu/ nr 2012-002849-38

## Introduction

Non-small cell lung cancer (NSCLC) is one of the most frequent cancer types and accounts for the highest cancer-related mortality worldwide [1–3]. Over the past decade, targeted therapies directed against oncogenic driver pathways have revolutionized the treatment of NSCLC tumours. One such targetable driver is the epidermal growth factor receptor (EGFR) pathway. Patients with an activating EGFR mutation are best treated with tyrosine kinase inhibitors (TKI), as they result in much higher response rates than other therapies such as chemotherapy or immunotherapy [4–8]. Therefore, EGFR TKI is the standard of care in EGFR mutation positive patients [9]. EGFR status is determined through a histological biopsy of the tumour [10]. However, obtaining a representative biopsy is not always feasible. This may be due to tumour localization making it difficult, if not impossible, to obtain a biopsy. Therefore, diagnosing TKI sensitive patients remains a challenge, highlighting the need for alternative (preferably non-invasive) means to identify them.

Positron emission tomography (PET) may provide such an alternative. Indeed, Bahce et al. demonstrated that EGFR mutation positive tumours could be identified using PET and [^11^C] erlotinib, a radiolabelled first generation EGFR TKI [11, 12]. In this study, [^18^F] afatinib, a radiolabelled second generation, irreversible EGFR TKI, was used as PET tracer. In mouse models with NSCLC cell lines (EGFR wild-type (A549) and EGFR mutated (HCC827) xenografts), Slobbe et al. showed accumulation of [^18^F]afatinib in NSCLC tumours [13, 14]. To date, however, no [^18^F]afatinib studies have been performed in patients.

The aim of the present study was to assess kinetics of [^18^F]afatinib in NSCLC patients and to derive the optimal tracer kinetic model for quantifying [^18^F]afatinib uptake in tumours. A secondary aim was to determine the simplified uptake parameter that best correlated with the preferred quantitative uptake parameter.

## Materials and methods

### Review medical ethics committee

This study was approved by the Medical Ethics Review Committee of the Amsterdam University Medical Center (location VUmc, Amsterdam, The Netherlands). Each patient gave written informed consent prior to inclusion.

### Patient inclusion

Ten patients with advanced stage NSCLC were enrolled. Although patients could have undergone treatment prior to inclusion, they all were afatinib naïve. An overview of patients included is provided in Table 1. A full list of in- and exclusion criteria can be found in Appendix 1.

**Table 1:** Baseline and scanning characteristics

Patient number	Gender F = female M = male	Age (y)	EGFR mutation present, y = yes n = no	[^18^F]Afatinib scanning protocol	[^15^O]H_2_O scanning protocol
1	M	68	Y	1	N
2	F	69	N	2	Y
3	M	51	Y	2	Y
4	M	69	Y	2	Y
5	F	57	Y	2	Y
6	F	53	Y	3	N
7	M	71	N	3	Y
8	M	71	N	3	Y
9	F	47	Y	3	Y
10	F	68	Y	3	Y

### [^18^F]Afatinib synthesis

Synthesis of [^18^F]afatinib was performed as described by Slobbe et al. [13]. Briefly, starting from 7-chloro-quinazoline-4(3H)-one, 3-chloro-4-trimethylammonium-nitrobenzene triflate was synthesized. Subsequently, 3-chloro-4-trimethylammonium-nitrobenzene triflate was labelled with fluorine-18 using the peptide coupling reagent benzotriazole-1-yl-oxy-tris-(dimethylamino)-phosphonium hexafluorophosphate (BOP). Finally, synthesized [^18^F]afatinib was purified by semipreparative HPLC chromatography .

### PET/CT scanning

The study comprised 3 scanning protocols. Each patient was scanned on an Ingenuity TF PET/CT (Philips, Best, the Netherlands). The first patient, included for dosimetry purposes, underwent 4 whole body scans following injection of 74 MBq [^18^F]afatinib. Each whole body scan (from top of the skull to mid-thighs to include all vital organs) consisted of 11 bed positions with 3 min per bed position. Scanning protocols 2 and 3 started with a low-dose CT scan, which was used for attenuation correction and image segmentation, followed by a 10-min [^15^O]H_2_O PET scan for assessment of perfusion. Four patients were scanned according to protocol 2, and 5 patients were scanned according to protocol 3. In the second sub-study (protocol 2), patients underwent a 90-min dynamic [^18^F]afatinib PET scan. In protocol 3, a [^15^O]H_2_O PET scan was followed by a low-dose CT and finally, a 60-min dynamic [^18^F]afatinib PET scan. All [^18^F]afatinib PET scans in protocols 2 and 3 were obtained after an intravenous bolus injection of 370 MBq (± 10%) [^18^F]afatinib. All [^15^O]H_2_O PET scans were obtained after an intravenous bolus injection of 370 MBq [^15^O]H_2_O.

- All PET emission scans were acquired in list mode and reconstructed retrospectively using a 3-dimensional row-action maximum-likelihood algorithm into time frames with progressive increase in frame duration [15]. Reconstructions included all usual corrections, such as detector normalization, and decay, dead time, attenuation, randoms, and scatter corrections. For [^15^O]H_2_O, 26 frames were used (1 × 10, 8 × 5, 4 × 10, 2 × 15, 3 × 20, 2 × 30 and 6 × 60 s). For the 90-min [^18^F]afatinib scan (protocol 2), 22 frames were used (1 × 15, 3 × 5, 3 × 10, 4 × 60, 2 × 150, 2 × 300 and 7 × 600 s). For the 60-min [^18^F]afatinib scan (protocol 3), 19 frames were used (1 × 15, 3 × 5, 3 × 10, 4 × 60, 2 × 150, 2 × 300 and 4 × 600 s).

### Blood sampling

In patients of protocols 2 and 3, both on-line (continuous) and manual (discrete) arterial sampling was performed during the dynamic [^18^F]afatinib PET scan; in protocol 2, sampling was only performed during the first dynamic scan. Manual samples were used to calibrate the on-line curve and to determine plasma to whole blood ratios together with fractions of labelled metabolites in plasma. In protocol 2, on-line sampling was performed for 40 min post injection (p.i.), at a rate of 5 mL/min for the first 5 min and 2.5 mL/min thereafter. In protocol 3, on-line sampling was performed for 20 min post injection (p.i.), again at a rate of 5 mL/min for the first 5 min and 2.5 mL/min thereafter. In all patients, manual arterial samples of 7 mL were taken at 5, 10, 15, 30, 40 and 60 min p.i. For protocol 2, a seventh sample was drawn at 75 min p.i.

### Metabolite analysis

Metabolites were analysed as described by Slobbe et al. [13]. Briefly, blood was collected in glass heparin tubes. Plasma was separated from blood cells, and activity concentrations were determined. Plasma was loaded onto a SepPak cartride, and polar metabolites were separated from non-polar metabolites. The non-polar fraction was analysed further by high-performance liquid chromatography (HPLC), finally resulting in plasma fractions of [^18^F]afatinib and its labelled metabolites.

### VOI definition

Each image is checked using the VINCI software (version 2.56.0) for patient movement or image artefacts. Motion correction is necessary if PET images do not fully overlap the CT scan that is done prior to scan acquisition. All volumes of interest (VOIs) were defined manually for each individual tumour using the software developed in-house. VOI definitions of tumours were based primarily on acquired CT images with images projected in parallel, avoiding necrosis and blood vessels as much as possible. If the shape of the tumour was erratic, a summed image of the last 4 frames was used to ensure correct definition of the VOI. If this PET image appeared to have relatively high uptake (visually) near the boundaries of a tumour, a 0.5-cm margin was added to the VOI. Definition of the image derived input function (IDIF) VOI within the descending aorta was performed in a standardized manner by drawing fixed VOIs in 10 slices of the descending thoracic aorta on a summed PET image of the first 6 frames, resulting in an overall VOI of 7.8 cm^3^ for all patients. VOIs were projected onto the dynamic PET images to extract time activity curves (TACs) for all regions.

### Input functions: metabolite and polar fraction correction

Conventional pharmacokinetic model analyses generally assume that only the unaltered parent tracer in plasma is capable of binding specifically to its target. Therefore, input functions were corrected for non-parent fractions, i.e. polar and non-polar metabolites, as it was not known whether polar fractions were metabolites or covalently bound/unavailable parent compounds.

The on-line arterial curve was calibrated using the manual arterial samples and extrapolated to the late manual samples (> 20 min p.i.) using an exponential fit (*y* = A*e^−Bt^ + C). Next, plasma to whole blood ratios and metabolite fractions obtained from the blood samples were fitted using a linear and a Hill-type function, respectively. Finally, a metabolite-corrected plasma curve was derived from the whole blood curve using the fitted plasma to whole blood ratios and metabolite fractions.

The IDIF was calibrated in a similar way as the on-line arterial curve. However, since IDIF data are obtained throughout the entire scan, extrapolation was not necessary.

To assess whether metabolite corrections are necessary, additional input functions were generated in which metabolite corrections were omitted (i.e. generating total sampler-based curves rather than metabolite-corrected sampler-based curves).

### Kinetic analysis

Analysis of [^18^F]afatinib tumour TACs was performed using 3 conventional pharmacokinetic plasma input models, all including an additional parameter for blood volume fraction (*V*_b_). A single-tissue compartment model (1T2k_Vb), an irreversible two-tissue compartment model (2T3k_Vb) and a reversible two-tissue compartment model (2T4k_Vb) were assessed [16]. All analyses were performed using both sampler-based and IDIF-based input functions.

Each model provides its own outcome measure. The main outcome measure for both single-tissue and reversible two-tissue compartment models is the volume of distribution (*V*_T_). *V*_T_ is defined for the single-tissue model as *K*_1_/*k*_2_ and for the 2T4K model as (*K*_1_/*k*_2_)∙(1 + *k*_3_/*k*_4_) and describes the equilibrium in- and efflux ratio of the tracer to and from the target tissue. Note that the initial assessment of the 2T4K model showed that BP_ND_ (=k_3_/k_4_) showed large variability and therefore, this parameter was not used. For the irreversible two-tissue model, the main outcome measure is the net influx rate constant *K*_i_ (=*K*1 × *k*3/(*k*2 + *k*3). K_i_ reflects the rate of (irreversible) tracer accumulation in the target tissue. Model fits were assessed using the Akaike information criterion (AIC) [17]. Results showing small differences in AIC were assessed also visually evaluated. Using this method, the optimal kinetic model was identified, and the outcome measure of this model was used to determine [^18^F]afatinib tumour uptake.

Tumour perfusion was derived from the [^15^O]H_2_O scans using the standard single-tissue compartment (1T2k_Vb) model combined with an IDIF [18]. By using the Pearson correlation between the kinetic parameter of interest of this model, F (blood flow) and the outcome measure of the [^18^F]afatinib PET scans, dependency of tumour tracer uptake on tumour perfusion was evaluated.

### Simplified measures

In order to assess whether simplification of scanning protocol and quantification of [^18^F]afatinib tumour uptake would be possible, several simplified measures were assessed, including standard uptake value (SUV) and tumour-to-blood ratio (TBR) [19]. SUV is defined as the radioactive concentration in the target tissue (C_image_) normalized by injected activity (IA) and bodyweight (BW) (SUV = C_image_∙BW/IA). TBR for both whole blood (TBR_WB) and plasma (TBR_PP: TBR parent plasma) and SUV was calculated, as well as for tumours at different time points (30–60 min p.i., 60–90 min p.i., total scan duration). Integrals of the TBR values were also assessed. Correlation of the optimal (kinetic uptake) outcome measure and these simplified measures was performed to evaluate which simplified measure best describes [^18^F]afatinib uptake. A list of simplified measures is given in Appendix 2.

### Statistical analysis

All kinetic analyses were performed using the software developed in-house. Descriptive statistical analyses were performed to assess and report endpoints. The Pearson correlation coefficient was used to analyse the correlation between on-line plasma input and IDIF input using the preferred parameter of interest obtained through pharmacokinetic modelling of [^18^F]afatinib. Correlation analysis using the Pearson correlation coefficient was also performed for the preferred parameter versus *K*_1_ of [^15^O]H_2_O as well as for K_1_ of the preferred pharmacokinetic model versus *K*_1_ of [^15^O]H_2_O to asses perfusion dependency. Correlation analysis for each simplified measure versus the preferred pharmacokinetic outcome measure was also performed using the Pearson correlation coefficient. Correlations were considered strong if the Pearson correlation coefficient was > 0.7 and very strong if it was > 0.9. Analyses were performed using IBM SPSS (version 22).

## Results

### Patient and scanning characteristics

Ten patients were included in this study. In 7 patients, an activating EGFR mutation was found in tumour DNA prior to inclusion. In the other patients, no mutation was found (EGFR wild type). No resistance mutations were found. Within the field of view of the scanner, a total of 16 tumours were identified. In patient 8, the start of arterial sampling was not captured by the on-line sampler, so the tumour TAC could only be analysed using IDIF. Therefore, 15 tumour VOIs were included in the analysis. Patient and PET scanning characteristics are given in Table 1, where also deviations from the scan protocol (i.e. missing scans) are indicated. The injected dose of [^15^O]H_2_O was 370 MBq for all patients. The injected dose of [^18^F]afatinib was 341 ± 37 MBq with a specific activity of > 18 MBq/μmol (i.e. 97 ± 55 MBq/μmol). There were no adverse or clinically detectable effects observed in any of the subjects.

### Sample data

**Fig. 1:**
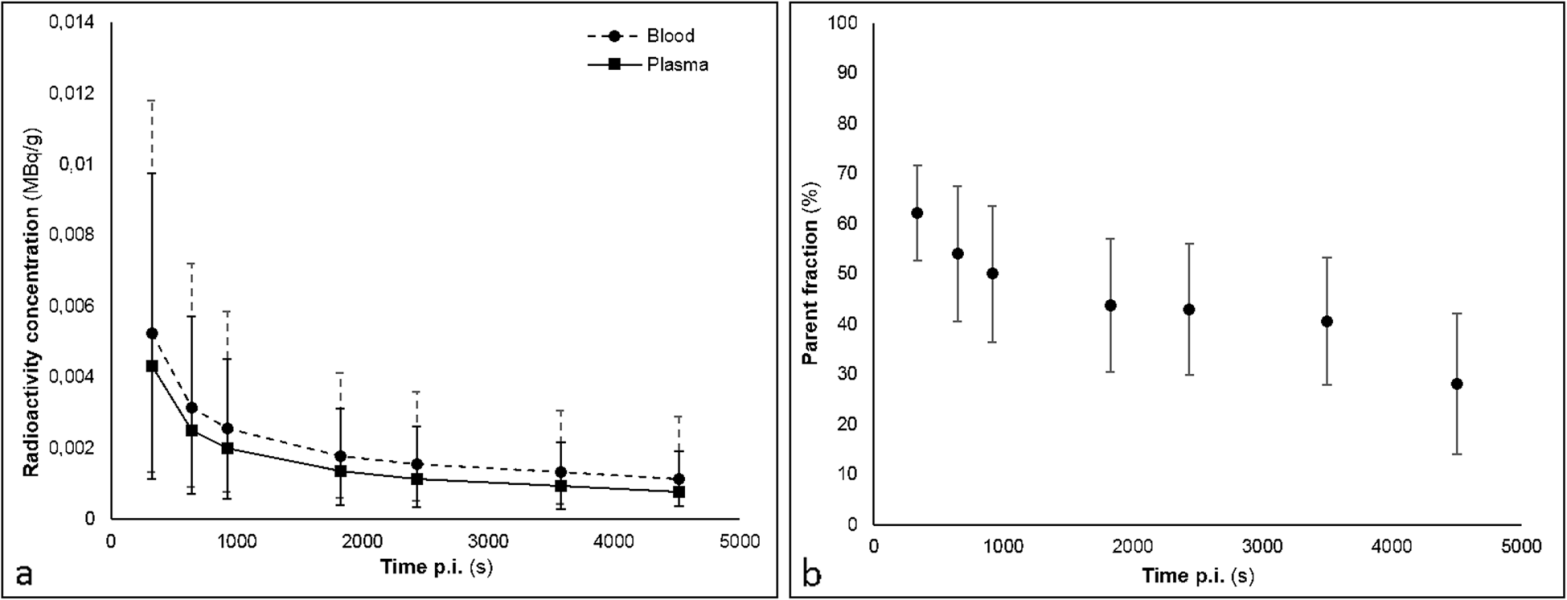
**a** Radioactivity concentrations of whole blood and plasma samples. **b** Parent fractions in plasma samples. Mean values are shown together with standard deviations (vertical bars)

Radioactivity concentrations of arterial whole blood and plasma samples are shown in Fig. 1a and plasma parent fractions in Fig. 1b.

### Pharmacokinetic model selection

**Fig 2:**
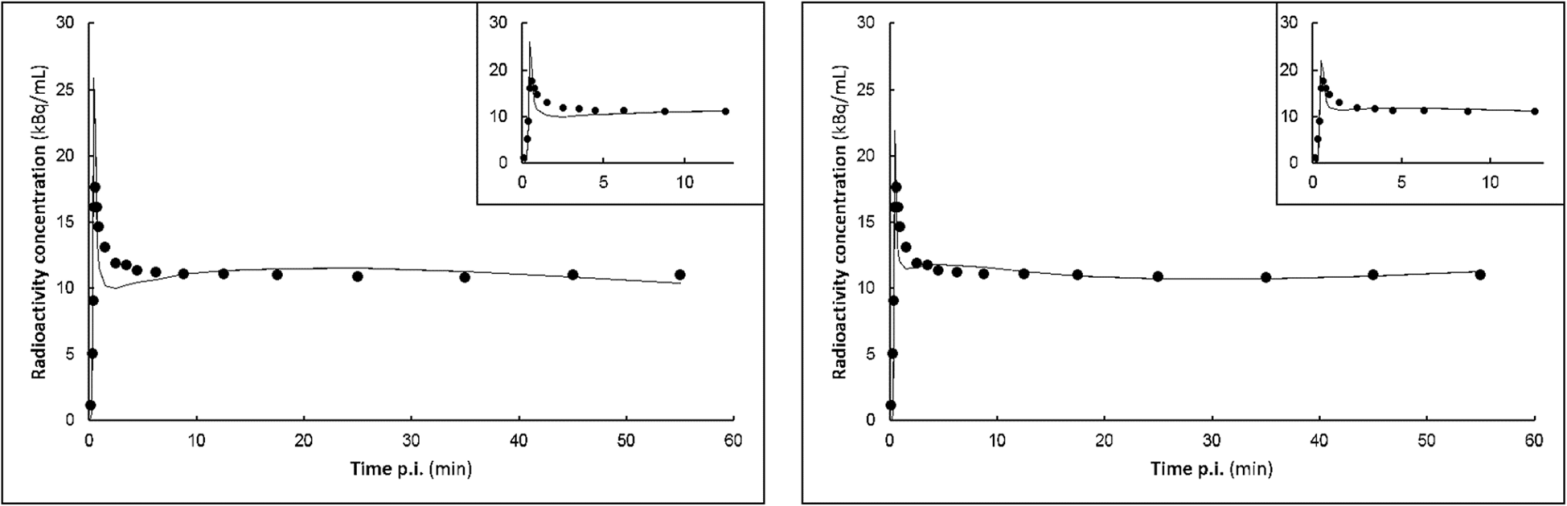
Fits of the tumour TAC of patient 7. Circles represent input data and curves model fits. Details of the fits of the peaks are shown as insets. **a** 1T2k_Vb. **b** 2T3k_Vb

Metabolite-corrected input functions using both sampler-based curves and IDIFs were created and used to fit the tumour TACs to the 3 conventional pharmacokinetic models. All fits were assessed for quality, both visually and using the AIC. Both indicated a preference for the 2T3k_Vb model (AIC 50/36% plasma/IDIF), followed by 1T2k_Vb (AIC 43/29%) and 2T4k_Vb (AIC 7/36%) models. In 2 patients, AIC indicated a preference for the 1T2k_Vb model, but subsequent visual assessment due to small (< 10%) AIC differences showed a similar or more probable fit of the 2T3k_Vb model. In Fig. 2, an example of such a discrepancy is shown. Therefore, the 2T3k_Vb model was used for analysing all tumour TACs.

### Correlation between sampler-based input function and IDIF

**Fig 3:**
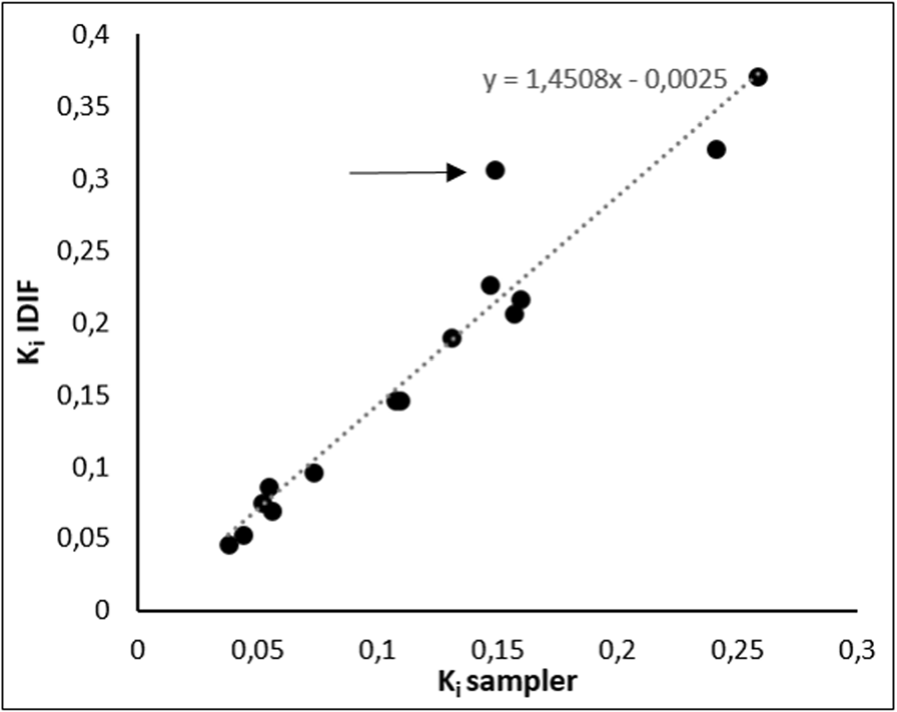
Correlation between [^18^F]afatinib *K*_i_ values obtained using on-line sampler and IDIF input functions

There was a very strong correlation between tumour *K*_i_ obtained using measured sampler-based input functions and those obtained using IDIFs (*r*^2^ = 0.93), as shown in Fig. 3. The outlier (indicated by the arrow in Fig. 3) corresponds with a patient where the 2T3K model was not the preferred model. Based on the correlation shown in Fig. 3, all subsequent analyses were performed using IDIF only.

### Input functions not corrected for metabolites

In protocol 2, tumour TACs were also fitted to the 3 models using input functions not corrected for metabolites to assess the necessity of metabolite correction. In 7 patients, visual assessment showed poorer quality of fits compared with fits obtained using metabolite-corrected plasma input functions (data not shown). The other 2 patients showed similar quality of fits. Corrections for labelled metabolites were therefore considered necessary and subsequent analyses were performed using metabolite-corrected input functions.

### Perfusion analysis

**Fig. 4:**
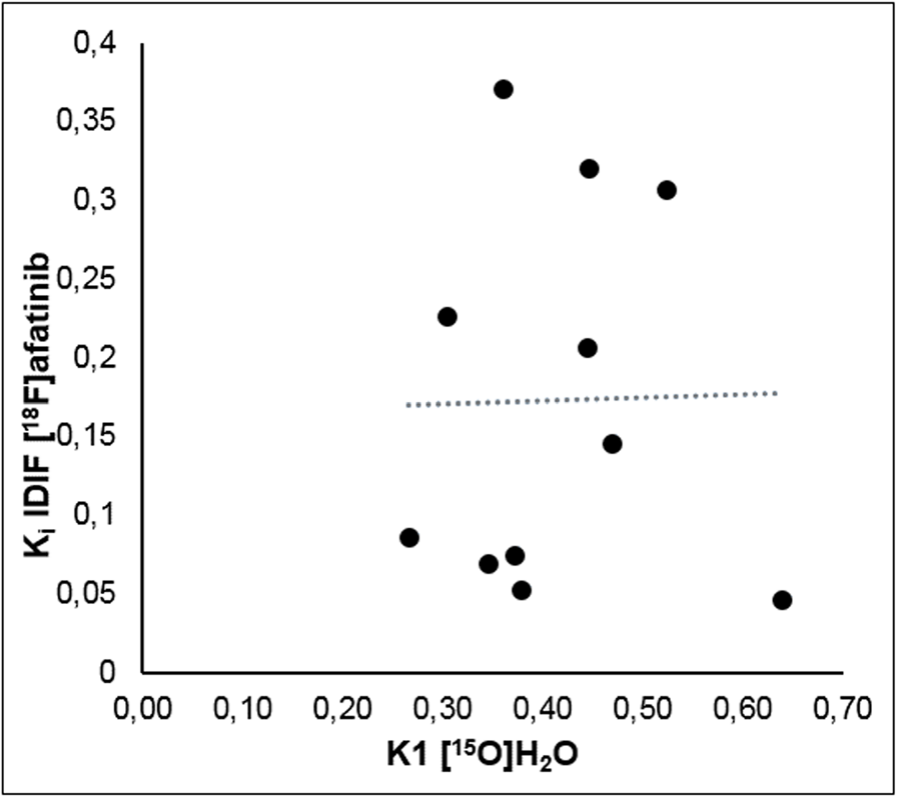
Correlation between *K*_i_ of [^18^F]afatinib (IDIF) and *K*_1_ of [^15^O]H_2_O

All except 2 patients underwent [^15^O]H_2_O scans. Clearly, the [^15^O]H_2_O PET scan was not part of the dosimetry protocol. In one patient, the [^15^O]H_2_O PET scan was cancelled due to time constraints. Blood flow in the tumour of patient 4 appeared to be unrealistically high (*K*_1_~1.9 mL/(min g)), presumably due to technical issues and patient motion. Therefore, all H_2_O data of this patient were excluded. No correlation between [^15^O]H_2_O *K*_1_ and [^18^F]afatinib *K*_i_ (*r*^2^ = 0.0003) was found, indicating that [^18^F]afatinib tumour uptake is not perfusion dependent, as shown in Fig. 4.

### Simplified measures

**Fig. 5:**
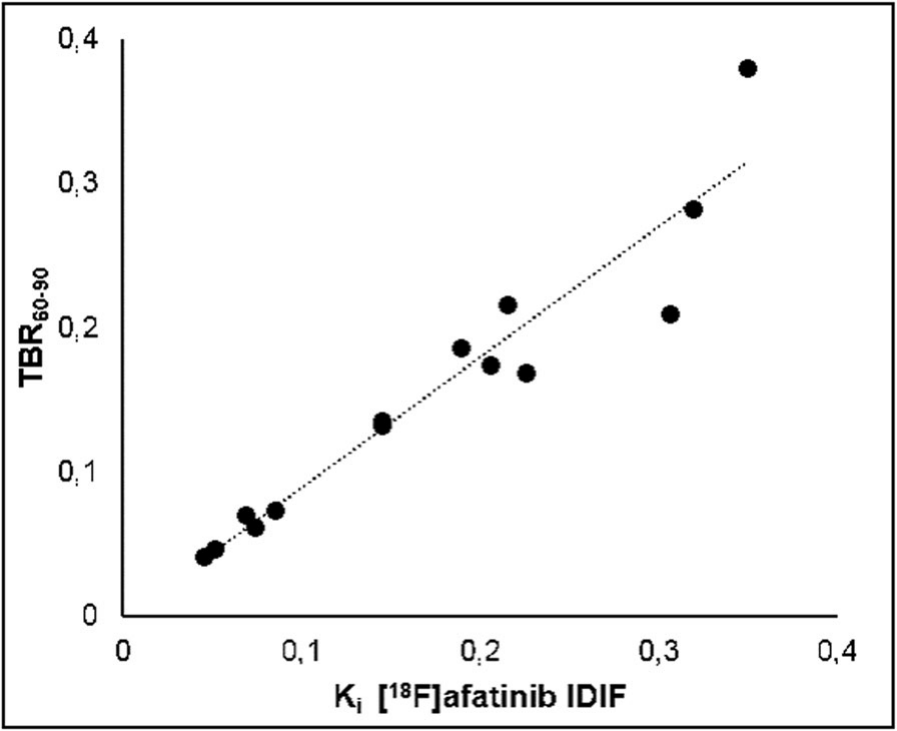
Correlation plot showing the correlation between *K*_i_ IDIF of [^18^F]afatinib and TBR_60–90_. *R*^2^ = 0.93

Analysis of several simplified measures showed that generally TBR based on metabolite-corrected plasma input generally performed better than TBR based on whole-blood input. TBR_60–90_ (tumour-to-blood ratio from 60 to 90 min) using plasma-based input-correlated best with [^18^F]afatinib *K*_i_ obtained using kinetic analysis (IDIF; *r*^2^ = 0.93), as shown in Fig. 5. This indicates that tumour uptake of [^18^F]afatinib can reliably be quantified using TBR_60–90_ as uptake measure.

All simplified measures, i.e. various SUV and TBR over other time intervals, and their correlations with *K*_i_ are given in Appendix 2. Every simplified measure shows a Pearson correlation coefficient of ≥ 0.75, indicating a strong to very strong correlation for each measure.

## Discussion

The aim of this study was to quantify [^18^F]afatinib tumour uptake in NSCLC patients. Dynamic scanning in combination with a metabolite-corrected sampler-based function was used to identify the tracer kinetic model that best describes [^18^F]afatinib tumour uptake. The irreversible two-tissue model showed the best fits, yielding *K*_i_ as parameter of interest. This result is in accordance with in vitro data, showing irreversible binding of afatinib to the EGF receptor [20].

### VOI definition

Pharmacokinetic modelling was performed on TACs derived from VOIs defined using both PET and (low-dose) CT images. Identifying blood vessels can be challenging on a low-dose CT, especially in hilar regions. In the delineation of tumour VOIs, we avoided large blood vessels; however, it is possible that some contribution of blood vessels was included in the VOIs located near these regions.

### Pharmacokinetic modelling using arterial sampling and IDIF

[^18^F]Afatinib *K*_i_ values obtained using metabolite-corrected IDIF correlated strongly with *K*_i_ values obtained using arterial input functions based on the on-line sampler. These results validate the use of an IDIF instead of an invasively measured input functions (arterial sampling). Since the on-line sampler was not used during the total duration of the scan, extrapolation was needed to create the sampler-based input functions, possibly leading to overestimation of the *K*_i_. In contrast, IDIF-based input functions are based on the full scan, which negates the need for extrapolation, producing more reliable input functions. Using IDIF instead of the on-line sampler allows for simplifying the scanning protocol in future studies. However, IDIF still needs to be corrected for metabolites. The parent fraction was around 70% after 5 min with a gradual decrease to approximately 30% after 75 min, indicating a rapid metabolism. In future studies, metabolite correction based on venous blood should be compared to metabolite correction using arterial blood to further simplify the scanning protocol (i.e. to omit the arterial cannula).

Considering that tumour uptake of [^18^F]afatinib is perfusion independent, the scanning protocol can be simplified by omitting the [^15^O]H_2_O perfusion scans. This allows for quantification of tumour [^18^F]afatinib uptake using a simplified measure.

The present results suggest that static whole body scanning is feasible using TBR_60–90_. This time interval of 60–90 min needs to be validated in larger studies.

### Clinical implications

Afatinib binds covalently to the kinase domain of the epidermal growth factor receptor. Affinity for this bond increases in the presence of an activating EGFR mutation [20]. Previous studies using radiolabeled [^11^C] erlotinib, a first-generation reversible EGFR TKI showed that uptake of [^11^C] erlotinib was significantly higher in tumours with an EGFR mutation [11]. Although these studies showed that PET was able to identify EGFR mutation positive tumours, the short half-life of ^11^C restricts the application of [^11^C] erlotinib in widespread clinical practice. By using a fluorine-18 tracer instead of a carbon-11 tracer, the problem of a short half-life can be addressed, thereby enabling its potential use in clinical practice.

The next important step for this tracer is to assess its capacity to predict the tumour EGFR mutational status, as this is a strong predictor for response to EGFR-directed TKI therapy, such as afatinib [8, 20, 21].

## Conclusion

Kinetics of [^18^F]afatinib in NSCLC tumours is best described by an irreversible two-tissue compartment model, where IDIF can be used rather than on-line arterial sampling. Tumour tracer uptake was shown to be perfusion independent. In this small series of patients, an excellent correlation was observed between TBR_60–90_ and *K*_i_ suggesting that [^18^F]afatinib can potentially be used in combination with (static) whole body scans.

## Data Availability

The datasets used and analysed during the current study are available from the corresponding author on reasonable request.

## Appendix 1

- Inclusion criteria

- Age 18–70
- Histologically proven NSCLC, with known EGFR mutational status (as determined by high resolution melting and DNA sequencing)
- Indication for afatinib therapy and planned to start treatment after scanning
- Life expectancy ≥ 12 weeks
- Malignant lesion of ≥ 1.5 cm within the chest as measured by CT
- Performance status Karnofsky ≥ 60%
- Written informed consent
  - Exclusion criteria
- Claustrophobia
- Pregnant or lactating patients
- Patients with a metal implant in the thorax that could cause attenuation artefact (e.g. pacemaker)
- Concurrent treatment with experimental drugs
- Anaemia
- Coumarin therapy

## Appendix 2

**Table 2:** Simplified measures

Simplified measure	Pearson correlation coefficient (measure vs. K_i_ IDIF)
TBR_WB (30–90 min)	0.87
TBR_WB (60–90 min)	0.79
TBR_PP (30–90 min)	0.87
TBR_PP (60–90 min)	0.87
SUV (30–90 min)	0.80
SUV (60–90 min)	0.78
TBR_integral_PP (30–90 min)	0.94
TBR_integral_PP (60–90 min)	0.93
TBR_integral_WB (30–90 min)	0.75
TBR_integral_WB (60–90 min)	0.75

*TBR* tumour-to-blood ratio, *WB* whole blood, *PP* parent plasma, *SUV* standardized uptake value
